# DNA Barcoding of *Prunus* Species Collection Conserved in the National Gene Bank of Egypt

**DOI:** 10.1007/s12033-022-00530-z

**Published:** 2022-08-13

**Authors:** Hossam A. Sayed, Shimaa Mostafa, Ibrahim M. Haggag, Neveen A. Hassan

**Affiliations:** National Gene Bank, Agricultural Research Center, B.O. 12619, Giza, Egypt

**Keywords:** DNA barcode, *Prunus* species, *Prunus armanica*, *Prunus persica*, *Prunus persica* var. nucipersica, *Prunus domestica* L., *P. salicina*

## Abstract

Two intergenic spacers cpDNA barcoding regions were used to assess the genetic diversity and phylogenetic structure of a collection of 25 *Prunus *accessions. The *trn*H-*psb*A and *trn*L-*trn*F intergenic spacers were able to distinguish and identify only four *Prunus* species. The average aligned length was 316–352 bp and 701–756 bp for *trn*H-*psb*A and *trn*L-*trn*F, respectively. The overall evolutionary divergence was higher in *trn*H*-psb*A than *trn*L*-trn*F. The transition/transversion bias (*R*) recorded as 0.59 in *trn*L*-trn*F and 0.89 in *trn*H*-psb*A. The number of invariable sites, nucleotide diversity (Pi), and the average number of nucleotide differences (k) was higher in the *trn*H-*psb*A region. The *trn*L-*trn*F records was above the other region in the number of variable sites, number of singleton variable sites, and the parsimony informative sites. Phylogenetic relationships among the 25 accessions of *Prunus* species were investigated. Most of the different *Prunus* species clustered in a homogenized distribution in both regions, except for the plum (*P. domestica*) accession (African Rose) was assigned with the peach *(P. persica)* accessions. The two intergenic cpDNA *trn*H-*psb*A and *trn*L-*trn*F were able to distinguish and identify the four *Prunus* species accessions.

## Introduction

The first crucial step in conserving plant genetic resources is the correct identification of the targeted species. A potential method to meet this identification is DNA barcoding, which is the identification of species by a short universal DNA sequence that exhibits a sufficient level of variation to discriminate among species [1, 2]. The emergence of DNA barcoding has had a positive impact on biodiversity classification and identification [3]. The primary goals of DNA barcoding technique are species identification of known specimens and discovery of overlooked species for enhancing taxonomy for the benefit of science and society [4]. Using DNA barcoding, a species can be identified from a tiny amount of tissue, seeds, or fragmentary materials [5]. After an extensive inventory of gene regions in the mitochondrial, plastid, and nuclear genomes of plants, four primary gene regions (*rbc*L, *mat*K, *trn*H-*psb*A, and ITS) have generally been agreed upon as the standard DNA barcodes of choice in most applications for plants [6–9]. Recently, research interest has spread through the DNA barcoding for economically important species of plants [10].

*Prunus* (or stone fruits) belongs to family *Rosacea*, is an economically important genus with approximately 200 species, grown in moderate regions [11]. The most common important cultivated species are; european plum (*P. domestica* L.), japanese plum (*P. salicina* Lindl*.*), sweet cherry *(P. avium* L.), sour cherry *(P. cerasus* L.), peach (*P. persica* (L.) Batsch), nectarine (*P. persica* var. nucipersica (Suckow) C. K. Schneid.), almond *(P. dulcis* (Mill.) D. A. Webb.), and apricot (*P. armeniaca* L.) [12]. *Prunus persica* includes peach and nectarine. The nectarine (*P. perscica var*. nucipersica) is a mutant strain of peach (*P. persica*), with special unique fruit characteristics [13]. *Prunus* genome is relatively small with about 250–300 Mbp [14]. The basic number of *Prunus* chromosomes is (x = 8). Almond (*P. dulcis*), peach (*P. persica*), apricot (*P. armeniaca* L.), sweet cherry (*P. avium* L.), Japanese plum (*P. salicina* Lindl.) are diploids (2*n* = 2 × = 16). Unless the European plum (*P. domestica* L.) is hexaploidy (2n = 6 × = 48), it is supposed resulted from the tetraploid species (*P. spinosa* L.) and the diploid species (*P. cerasifera* Ehrh.) [15]. The correct identification and characterization of plant genetic resources (PGR) is important for germplasm utilization [16]. Using modern DNA-based markers is necessary for gene bank management [17].

The overall goal of this study is to assess the genetic diversity and phylogenetic structure of a collection of 25 *Prunus* accessions grown in Egypt conserved in the National Gene Bank, utilizing two intergenic DNA barcoding regions (*trn*H-*psb*A and *trn*L-*trn*F).

## Materials and Methods

### Plant Materials

The current research conducted using 25 *Prunus* genotypes belonging to 5 species grown in Egypt, collected from different locations. The twenty-five *Prunus* accessions were collected, conserved, and maintained in the gene bank greenhouses. The samples used in this study are demonstrated in Table 1.

**Table 1:** *Prunus* species and cultivar/variety name of *Prunus* accession samples

*Prunus* species	Accessions sample name
Almond (*Prunus dulcis* (Mill.))	Sweet almond, old-local cultivar “Hash”
	Sweet almond, old-local cultivar “Adm”
	Sweet almond, local variety
	Bitter almond, local variety
Apricot (*Prunus armeniaca* L.)	Old-local variety “Ammar01-clone1”
	Old-local variety “Ammar02-clone2”
	Commercial variety “Hammway”
	Commercial local variety “El-Amal”
	Commercial local variety “Hayed”
	Commercial variety “Canino”
Peach (*Prunus persica* (L.) Batsch)	Old-local variety “Balady”
	Old-local variety “Mit Ghamar”
	Commercial variety “Early Grand”
	Commercial variety “Early Swelling”
	Commercial variety “Desert Red”
	Commercial variety “Florida Prince”
Nectarine (*Prunus persica var*. nucipersica (Suckow) C. K. Schneid.)	Commercial variety
European plum (*Prunus domestica* L.)	Old-local variety “Succari”
	Old-local variety “Bokra”
	Commercial variety “Hollywood”
	Commercial variety “Santa Rosa”
	Commercial variety “Pioneer”
	Commercial variety “African Rose”
	Commercial variety “English”
Japanese plum (*Prunus salicina* Lindl.)	Commercial variety “Japanese”

### DNA Isolation, PCR Thermocycling Profile of *Prunus* DNA Barcoding Identification

The genomic DNA (gDNA) of the samples was extracted using Qiagen DNeasy kit (cat No. 69104). The DNA was quantified using NanoDrop™ OneC (cat No. 840-329700) and adjusted to 50 ng/µl and used in the reactions. The twenty-five different *Prunus* samples were identified using two chloroplast DNA intergenic regions (*trn*H-*psb*A and *trn*L-*trn*F). The PCR reaction amplifications were performed on BioRad™ T100 thermal Cycler (No. 1861096), in 25 µl reaction volume, containing 2X of EmeraldAmp® MAX PCR mix (RR320A), 50 ng gDNA, and 20pMol for each primer. The primer sequence and thermocycling profile of PCR are demonstrated in Table 2.

**Table 2:** DNA chloroplast region, primer name and sequence, PCR thermocycling profile, and reference

DNA chloroplast region	Primer forward name and sequence	Primer reverse name and sequence	PCR thermocycling profile	Reference
*trn*H-*psb*A	*trn*H^GUG^: CGCGCATGGTGGATTCACAATCC	*psb*A: GTTATGCATGAACGTAATGCTC	94 °C for 3 min, 34 cycles (94 °C for 30 s, 50 °C for 2 min, 72 °C for 5 min), and final extension for 5 min	[18]
*trn*L-*trn*F	*trn*-c: CGAAATCGGTAGACGCTACG	*trn*-f: ATTTGAACTGGTGACACGAG	94 °C for 3 min, 34 cycles (94 °C for 30 s, 61.2 °C for 2 min, 72 °C for 5 min), and final extension for 5 min	[19]

DNA sequencing was carried out by Potsdam, Institute of Biochemistry and Biology (Potsdam, Germany) using an ABI sequencer. All sequences were submitted to NCBI GenBank, USA. GenBank provided accession numbers for the nucleotide sequences of each accession for each of the two loci, as demonstrated in Table 3.

**Table 3:** *Prunus* accessions name, Genbank accession numbers for the for the two barcoding loci (*trn*H-*psb*A and *trn*L-*trn*F)

No	*Prunus* species	*Prunus* accessions name	NCBI Genbank accession number	
			*trn*H*-psb*A	*trn*L-*trn*F
1	Almond (*P. dulcis* (Mill.))	Sweet almond, old-local cultivar “Hash”	OM328809	OM720097
2		Sweet almond, old-local cultivar “Adm”	OM328810	OM720098
3		Sweet almond, local variety	OM328811	OM720099
4		Bitter almond, local variety	OM328812	OM720100
5	Apricot (*P. armeniaca* L.)	Old-local variety “Ammar01-clone 1”	OM416742	OM720101
6		Old-local variety “Ammar02-clone 2”	OM416743	OM720102
7		Commercial variety “Hammway”	OM416744	OM720103
8		Commercial local variety “El-Amal”	OM416745	OM720104
9		Commercial local variety “Hayed”	OM416746	OM720105
10		Commercial variety “Canino”	OM416747	OM720097
11	Peach (*P. persica* (L.) Batsch)	Old-local variety “Balady”	OM416748	OM720106
12		Old-local variety “Mit Ghamar”	OM416749	OM720107
13		Commercial variety “Early Grand”	OM416750	OM720108
14		Commercial variety “Early Swelling”	OM416751	OM720109
15		Commercial variety “Desert Red”	OM416752	OM720110
16		Commercial variety “Florida Prince”	OM416753	OM720111
17	Nectarine (*P. persica var. nucipersica* (Suckow) C. K. Schneid.)	Nectarine, commercial variety	OM416754	OM720112
18	European plum (*P. domestica* L.)	Old-local variety “Succari”	OM416755	OM720113
19		Old-local variety “Bokra”	OM416756	OM720114
20		Commercial variety “Hollywood”	OM416757	OM720115
21		Commercial variety “Santa Rosa”	OM416759	OM720117
22		Commercial variety “Pioneer”	OM416760	OM720118
23		Commercial variety “African Rose”	OM416761	OM720119
24		Commercial variety “English”	OM416762	OM720120
25	Japanese plum (*P. salicina* Lindl.)	Commercial variety “Japanese”	OM416758	OM720116

### The Sequences Alignment and Phylogenetic Trees

The sequences of *trn*H*-psb*A and *trn*L*-trn*F for the two loci were subjected to NCBI–BLASTN online tool http://blast.ncbi.nlm.nih.gov/Blast.cgi [20] to check the sequence similarity against sequences in the nucleotide collection (nr/nt) database. BLASTN default parameters were used and the organism selected was *Prunus* species in this database. Alignments of sequence were achieved by MUSCLE algorithm [21]. The evolutionary rate parameters, the pattern of nucleotide substitutions, and the average of evolutionary divergence over all the sequences, and phylogenetic trees were generated based on the Maximum Likelihood (ML) model, using MEGA version 11 software [22], other parameters of sequence diversity were calculated using DnaSP version5 [23].

## Results and Discussion

The average aligned length was 316–352 bp and 701–756 bp, for *trn*H*-psb*A and *trn*L*-trn*F loci, respectively. The *trn*H*-psb*A over all evolutionary divergence was higher (0.05) than in *trn*L*-trn*F (0.007). The transition/transversion bias (*R*) recorded as 0.59 and 0.89 in *trn*L*-trn*F and *trn*H*-psb*A, respectively.

The number of invariable sites was higher in *trn*H-*psb*A than in *trn*L-*trn*F (670 and 214, respectively). While, the number of variable (polymorphic) and singleton variable sites were lower (18 and 6) in *trn*H-*psb*A than in the other loci (77 and 47). The nucleotide diversity (Pi) and the average number of nucleotide differences (k) in *trn*H-*psb*A was lower than the other region. Meanwhile, the number of parsimony informative sites was higher (30) in *trn*L-*trn*F than the other region (12), Table 4 represent the results.

**Table 4:** Nucleotide sequence parameters for *trn*H-*psb*A and *trn*L-*trn*F regions, based on calculations of DnaSP-5 software

Sequence parameter	*trn*H-*psb*A	*trn*L-*trn*F
Number of invariable (monomorphic) sites	670	214
Number of variable (polymorphic) sites	18	77
Number of singleton variable sites	6	47
Number of parsimony informative sites	12	30
Sequence conservation (C)	0.967	0.695
Nucleotide diversity (Pi)	0.00592	0.03652
Average number of nucleotide differences (k)	4.070	10.627

### *trn*H-*psb*A Loci Sequence Analyses

The *trn*H*-psb*A loci length across the twenty-five *Prunus* accessions ranged from 316 to 352 bp. The nucleotide frequencies for A, T, C and G was 37.6%, 37.6%, 12.4% and 12.4%, respectively. The rate of different transitional substitutions from G to A was equal to those from C to T (16.73). On the other hand, the transversionsal substitution rates was equal as it recorded 10.44 for transversion from T to A, from C to A, and from G to T. While, it reached 3.44 in transversion from G to C, results shown in Table 5.

**Table 5:** ML estimate of the pattern of nucleotide substitution for *trn*H*-psb*A loci sequences across the twenty-five *Prunus* accessions, as calculated by MEGA version 11

	A	T	C	G
A	–			
T	*10.44*			
C	*10.44*	**16.73**	–	
G	**16.73**	*10.44*	*3.44*	–

Each entry is the probability of substitution (*r*) from one base (row) to another base (column). Rates of different transitional substitutions are shown in bold and those of transversionsal substitutions are shown in italics. Substitution pattern and rates were estimated under the Tamura (1992) model (+ G). A discrete Gamma distribution was used to model evolutionary rate differences among sites. Evolutionary analyses were conducted in MEGA11

### *trn*H-*psb*A Phylogenetic Tree

**Fig. 1:**
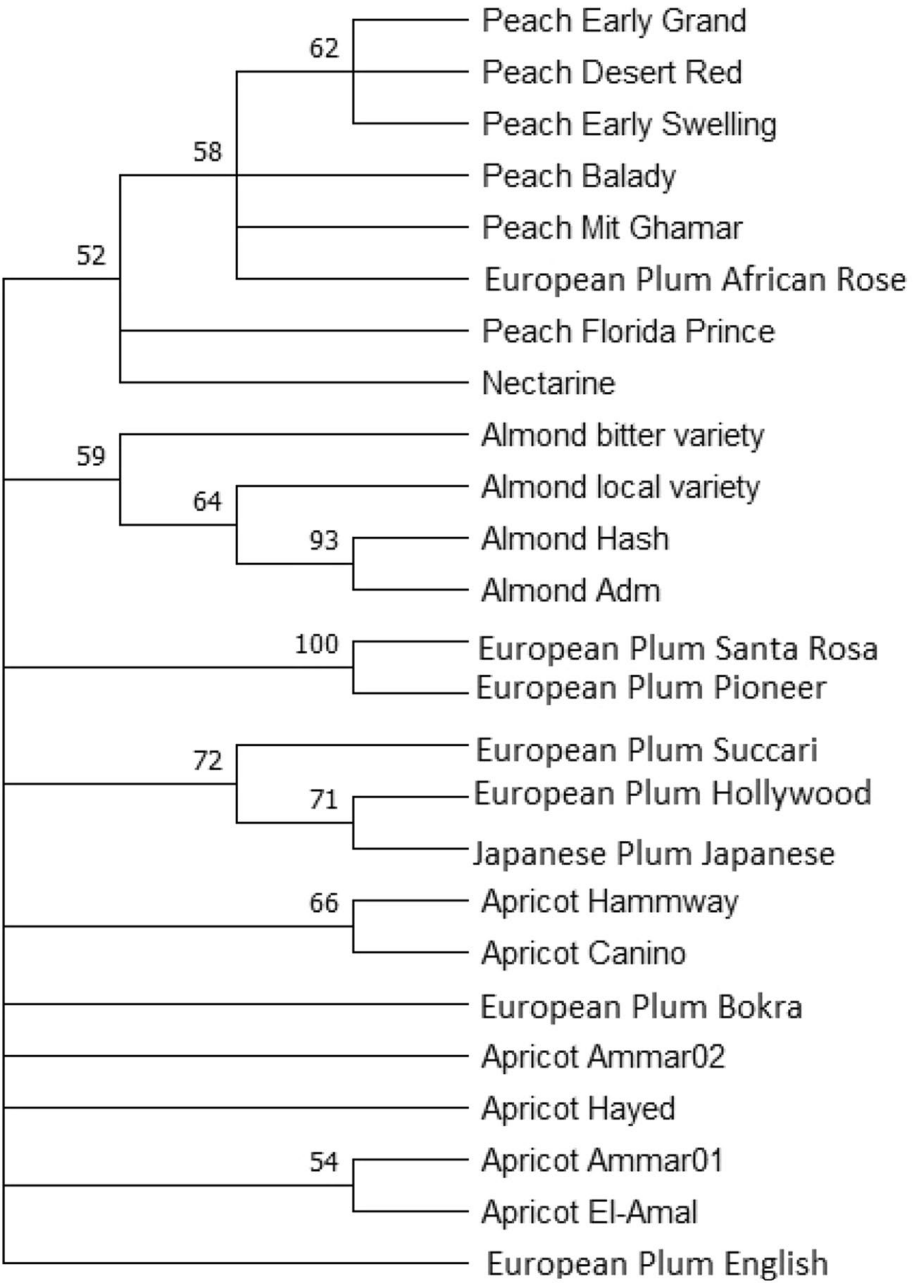
ML phylogeny tree based on *trn*H*-psb*A sequences, showing the relationships among the twenty-five *Prunus* accessions*.* Bootstrap values were indicated for each node (500 replicates), cut-off value for consensus tree is 50%, as calculated by MEGA version 11

The phylogentic tree computed from the *trn*H*-psb*A chloroplast region (Fig. 1) for the different *Prunus* species, assigned the peach, almond, and apricot to its relative species.

The Japanese plum accession (*P. salicina*) was assigned among the European plum (*P. domestica*) species accessions in the phylogenetic tree. European plum accessions (Bokra, and English) were clustered away from the related species accessions. Also, African Rose European plum accession was clustered among the peach accessions. Almond (*P. dulcis*) samples were homogenized and grouped together in the same group, where the two local accessions (Hash and Adm) were closer to each other than the other two samples. Peach (*P. persica*) and Nectarine (*P. persica var*. nucipersica) were grouped in the similar group. The apricot (*P. armeniaca*) accessions (Hammaway and Canino) constructed together, as they were closer to each other than the other accessions.

### *trn*L-*trn*F Region Sequence Analyses

The *trn*L*-trn*F chloroplast region length across the different *Prunus* sequences length ranged from 701 to 756 bp. The nucleotide frequencies for *trn*L*-trn*F region sequence was as equal for T and A (32.99%), and equal in G and C as 17.01%. The lowest rate of transitional substitution events was 5.14 for transition substitution from G to C. While, it was equal rate (9.98) in the transition substitution from T to A, from C to A, and from G to T. The transversion substitution from C to T had the equal value (13.04) as for the value of transversion from G to A (results shown in Table 6). The estimates of average evolutionary divergence over all sequences for *trn*L*-trn*F region was 0.007.

**Table 6:** ML estimate of the pattern of nucleotide substitution for *trn*L*-trn*F loci sequences across the twenty-five *Prunus* accessions, as calculated by MEGA version 11

	A	T	C	G
A	–			
T	*9.98*	–		
C	*9.98*	**13.04**	–	
G	**13.04**	*9.98*	*5.14*	–

Each entry is the probability of substitution (r) from one base (row) to another base (column). Rates of different transitional substitutions are shown in bold and those of transversionsal substitutions are shown in italics. Substitution pattern and rates were estimated under the Tamura (1992) model (+ G). A discrete Gamma distribution was used to model evolutionary rate differences among sites. Evolutionary analyses were conducted in MEGA11

### *trn*L-*trn*F Phylogenetic Tree

**Fig. 2:**
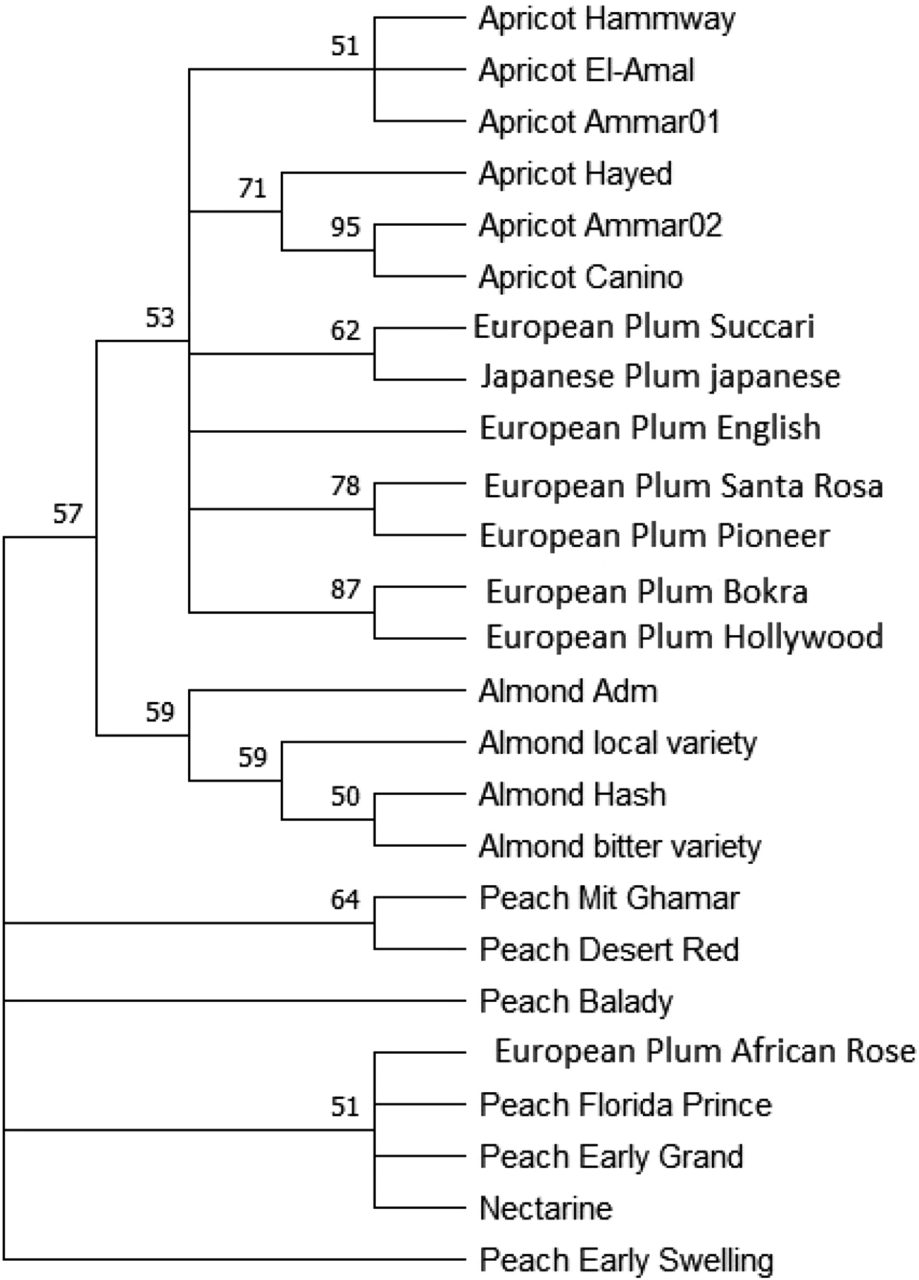
ML phylogenetic tree based on *trn*L*-trn*F region sequences, showing the relationships among the twenty-five *Prunus* accessions*.* Bootstrap values are indicated for each node (500 replicates), cut-off value for consensus tree is 50%, as calculated by MEGA version 11

The *trn*L-*trn*F-based phylogenetic (Fig. 2) clustered most the *Prunus* species properly, with two exceptions. First: the African Rose European plum accession, was clustered distantly away from related species near to peach species (*P. persica*) accessions*.* Second: the Japanese plum (*P. salicina*) was assigned amomg the European plum (*P. domstica*) species accessions. The apricot (*P. armeniaca*) accessions clustered together in two groups, as accessions (Hammway, El-Amal and Ammar01) clustered in the first group, while accessions (Hayed, Ammar02, and Canino) clustered in the second. The European plum (*P. domestica*) species accessions were clustered in a homogenized groups, except the Japanese plum accession (*P. salicina*) was assigned with the succari European plum species accession. The almond (*P. dulcis*) species accessions were grouped together in a related cluster. The peach (*P. persica*) and nectarine (*P. persica var.* nucipersica) accessions were grouped in a related groups, where the nectarine (*P. persics var*. nucipesica) acession was in the same group with Florida Prince, and Early Grand peach. the two accessions (Balady and Early Swelling) were clustered in a distant groups. The African Rose (European plum) species accession was clustered in the same group with Florida Prince, Early Grand, and Nectarine peach accessions.

## Concatenated Sequences-Based Phylogenetic Tree

The concatenated (combined) sequences were assembled and aligned from *trn*H*-psb*A and *trn*L*-trn*F sequences for the twenty-five *Prunus* accessions.

**Fig. 3:**
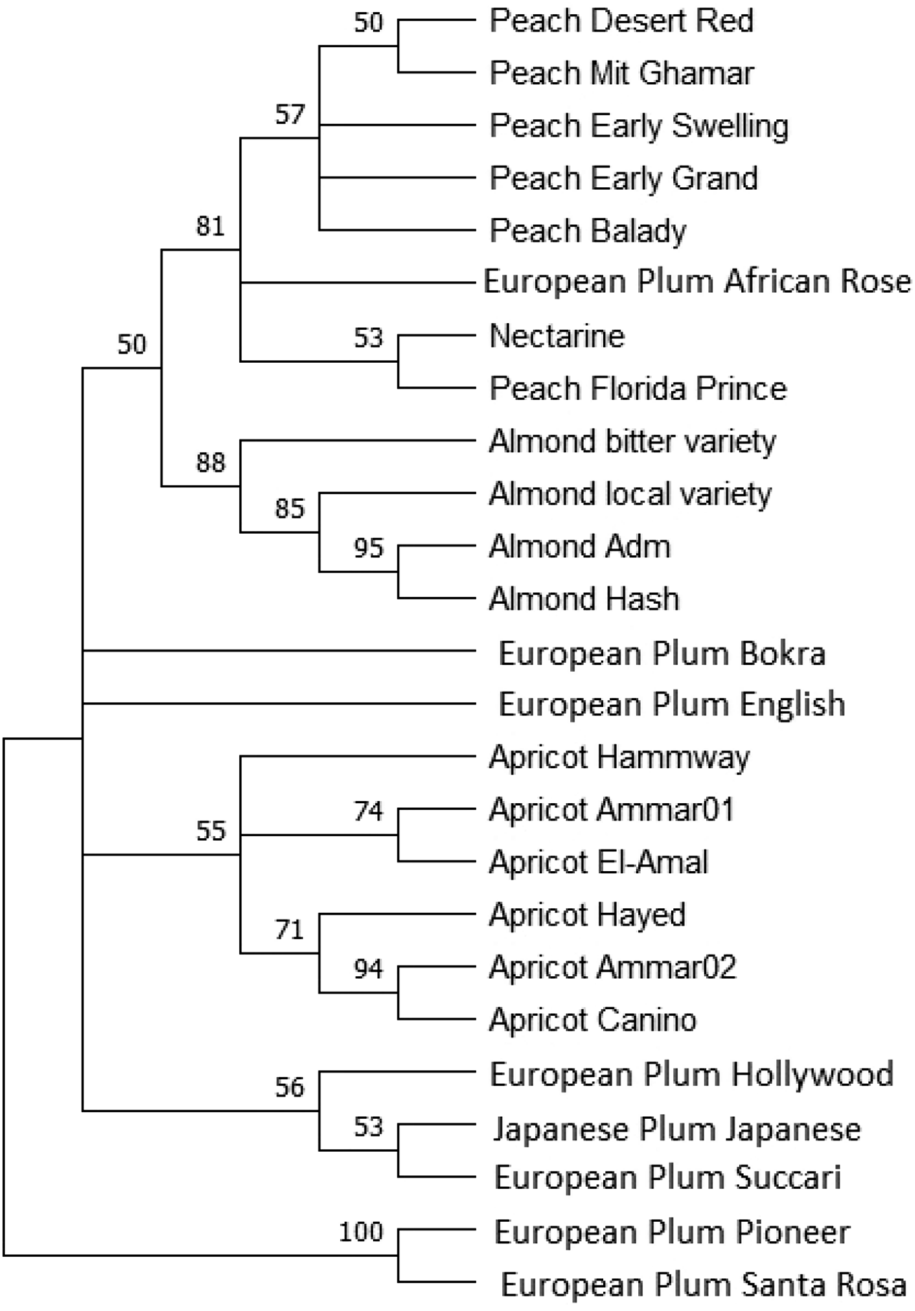
ML phylogenetic tree based on the concatenated sequences of both *trn*H*-psb*A and *trn*L*-trn*F sequences, showing the relationships among the twenty-five *Prunus* species*.* Bootstrap values are indicated for each node (500 replicates), cut-off value for consensus tree is 50%, as calculated by MEGA version 11

The concatenated-based phylogentic tree (Fig. 3) demonstrated an overview for the combined sequences of the two chloroplast intergenic regions across the five *Prunus* species for the 25 *Prunus* accessions. The most noted observation was that most *Prunus* species clustered together with the same relative species. Except, the European plum (*P. domestica*) accession (African Rose) which was assigned with the peach (*P. persica*) accessions. Also, the Japanese plum (*P. salicina*) accession assigned with the European plum (*P. domestica*) accessions.

The two accessions of European plum (Bokra and English) grouped away from the other relative European plum accessions, as thesse accessions were used only for pollination not for commercial purposes. The almond *(P. dulcis*) accessions were clustered together, as the local accessions (Adm and Hash) were near to each other. The apricot (*P. armeniaca*) accessions samples were clustered together at the same group. The peach (*P. persica*) and the nectarine (*P. persica var*. nucipersica) accession samples were related to each other.

Teberlet et al. [24] proposed six primers for three non-coding chloroplast regions. These primers were tested and reused as universal primers for wide range of taxonomic plant groups. These regions were latter used by many researchers to investigate the systematics and phylogentic relationships of *Prunus* species [18, 19, 25, 26]. Meanwhile, Uncu [27] used *trn*H-*psb*A region sucessefully to detect the fraud of apricot kernels to the almond valuable oil.

In the present study, the intergenic chloroplast regions *trn*L^UAA^-*trn*F^GAA^ and *trn*H-*psb*A, which was first proposed by Teberlet et al. [24], were able to identify the different *Prunus* species, and were able to characterize the different accessions. The *trn*L-*trn*F region had higher values in number of polymorphic sites, number of singleton variable sites, number of parsimony informative sites, nuclotide diversity, and average number of nucleotide differences. Meanwhile, *trn*H-*psb*A had evolutionary divergence, transition/transversion bias, monomorphic sites, and sequence conservation values higher than the second region.

The two intergenic regions were able to identify only four species, and were not able to identify *P. salicina* species, as *P. salicina* species was assigned with *P. domestica* species. The most notable observation in the phylogentic clusters was that the African Rose European plum accession, was distantly away from the related species, near to peach species accessions. Since this accession breeding ancestors had peach parents (data not published). The Japanese plum accession (*P. salicina*) is less resolved here as it was assigned among the European plum species (*P. domestica*) accessions, it could be for the selections proceeded for this adapted old-local variety. The nectarine accession (*P. persica var*. nucipersica) was assigned properly with peach species (*P. persica*) accessions, as nectarine is a mutant strain of peach [13]. It was observe that across the three constructed phylogenetic trees that almond (*P. dulcis*) and peach (*P. persica*) is closer to each other, as it was evolutionary hybridized [28]. Bortiri et al. [25] used *trn*L-*trn*F regions to identify different *Prunus* species, indicated little variations because of the monophyletic divergence of *Prunus*. Batnini et al. [26] used *trn*L-*trn*F and *trn*H-*psb*A regions in studying the genetic diversity among different *Prunus* species, resulting in high variability among studied species, with higher average than our obtained results.

## Conclusion/Future Perspectives

The current research constructed the phylogentic relationships of *Prunus* collection. This step is a cornerstone in identifying the conserved *Punus* germplasm, which will help in the crop development, sustainable use and impeovement of *Prunus*.
